# High Prevalence of Polyclonal *Plasmodium falciparum* Infections and Association with Poor IgG Antibody Responses in a Hyper-Endemic Area in Cameroon

**DOI:** 10.3390/tropicalmed8080390

**Published:** 2023-07-29

**Authors:** Marie Florence A Bite Biabi, Balotin Fogang, Estelle Essangui, Franklin Maloba, Christiane Donkeu, Rodrigue Keumoe, Glwadys Cheteug, Nina Magoudjou, Celine Slam, Sylvie Kemleu, Noella Efange, Ronald Perraut, Sandrine Eveline Nsango, Carole Else Eboumbou Moukoko, Jean Paul Assam Assam, François-Xavier Etoa, Tracey Lamb, Lawrence Ayong

**Affiliations:** 1 Malaria Research Unit, Centre Pasteur du Cameroun, Yaounde BP 1274, Cameroon; bite_marie@yahoo.fr (M.F.A.B.B.); b.fogang@yahoo.fr (B.F.); essanguiestelle@yahoo.fr (E.E.); malobafranklin@gmail.com (F.M.); donkeu.josiane@gmail.com (C.D.); rkeumoe@gmail.com (R.K.); glwadys2011@gmail.com (G.C.); mapejeni@gmail.com (N.M.); kemleufr@yahoo.fr (S.K.); noella.efange@yahoo.com (N.E.); nsango2013@yahoo.fr (S.E.N.); elsecarole@yahoo.fr (C.E.E.M.); 2 Department of Biochemistry, Faculty of Science, University of Douala, Douala BP 2701, Cameroon; 3 Department of Animal Biology and Physiology, Faculty of Science, University of Yaounde I, Yaounde BP 812, Cameroon; 4 Department of Biological Sciences, Faculty of Medicine and Pharmaceutical Sciences, University of Douala, Douala BP 2701, Cameroon; 5 Department of Biochemistry, Faculty of Science, University of Yaounde I, Yaounde BP 812, Cameroon; 6 Department of Medical Laboratory Sciences, Faculty of Science, University of Buea, Buea BP 63, Cameroon; 7 Department of Pathology, School of Medicine, University of Utah, 15 N Medical Drive, Salt Lake City, UT 84112, USA; celine.slam@biochem.utah.edu; 8 Department of Biochemistry, Faculty of Science, University of Buea, Buea BP 63, Cameroon; 9 Centre Pasteur du Cameroun Annex, Garoua BP 921, Cameroon; perraut@pasteur-yaounde.org; 10 Department of Microbiology, Faculty of Science, University of Yaounde I, Yaounde BP 812, Cameroon; assamjean@yahoo.fr (J.P.A.A.); fxetoa@yahoo.fr (F.-X.E.)

**Keywords:** *Plasmodium falciparum*, polyclonal infection, adaptive immune responses

## Abstract

Malaria remains a major public health problem worldwide, with eradication efforts thwarted by drug and insecticide resistance and the lack of a broadly effective malaria vaccine. In continuously exposed communities, polyclonal infections are thought to reduce the risk of severe disease and promote the establishment of asymptomatic infections. We sought to investigate the relationship between the complexity of *P. falciparum* infection and underlying host adaptive immune responses in an area with a high prevalence of asymptomatic parasitaemia in Cameroon. A cross-sectional study of 353 individuals aged 2 to 86 years (median age = 16 years) was conducted in five villages in the Centre Region of Cameroon. *Plasmodium falciparum* infection was detected by multiplex nested PCR in 316 samples, of which 278 were successfully genotyped. Of these, 60.1% (167/278) were polyclonal infections, the majority (80.2%) of which were from asymptomatic carriers. Host-parasite factors associated with polyclonal infection in the study population included peripheral blood parasite density, participant age and village of residence. The number of parasite clones per infected sample increased significantly with parasite density (r = 0.3912, *p* < 0.0001) but decreased with participant age (r = −0.4860, *p* < 0.0001). Parasitaemia and the number of clones per sample correlated negatively with total plasma levels of IgG antibodies to three highly reactive *P. falciparum* antigens (MSP-1p19, MSP-3 and EBA175) and two soluble antigen extracts (merozoite and mixed stage antigens). Surprisingly, we observed no association between the frequency of polyclonal infection and susceptibility to clinical disease as assessed by the recent occurrence of malarial symptoms or duration since the previous fever episode. Overall, the data indicate that in areas with the high perennial transmission of *P. falciparum*, parasite polyclonality is dependent on underlying host antibody responses, with the majority of polyclonal infections occurring in persons with low levels of protective anti-plasmodial antibodies.

## 1. Introduction

Malaria remains a major public health threat, with morbidity and mortality rates on the rise in several parts of the world, as revealed in the latest editions of the World Malaria Report [1,2,3]. Efforts to eliminate the disease are hampered, in particular by the increasing spread of insecticide and antimalarial drug resistance [2,4,5,6], exacerbated by the complexity of malaria parasite populations and the lack of a broadly effective vaccine [7,8,9]. Host responses to natural infection are often not sufficiently protective, given their slow development rates, short half-lives and allele-specificity [10,11,12,13,14,15]. It is known that repeated infection with malaria parasites or subclinical carriage of parasites for long durations can result in sustained natural antimalarial immunity and that infection with multiple parasite clones can increase the breadth, especially of adaptive antibody responses, leading to stronger protection against subsequent malaria [16,17,18,19,20]. Multiclonal infections originate either from co-transmission by mosquito vectors of genetically different parasite clones in a single blood meal (co-infections) or from overlapping infection in cases of repeated exposure to the bite of infected mosquitoes (superinfections) [21,22]. Indeed, in areas of high perennial transmission of the most deadly form of malaria parasites, *P. falciparum*, infection with multiple clones of the parasite is common, as well as a high prevalence of asymptomatic parasitaemia [23]. Whether multiclonal infections occur as a result of waning natural immunity that allows for the expansion of multiple genetically distinct parasite clones or due to the introduction of new parasite populations in the community remains not well understood. Therefore, understanding the association between multiclonal infection and host immune responses may be essential for predicting the risk of clinical malaria and monitoring success of malaria control interventions. 

In this study, we sought to assess the prevalence and the host and parasite factors associated with multiclonal *P. falciparum* infection in an area of high perennial transmission and high prevalence of asymptomatic malaria parasitaemia in Cameroon, one of 11 “high burden high impact” countries that accounted for 68% of the global estimated malaria cases and 70% of global estimated deaths in 2021 [3]. The country can be stratified into three zones of varying malaria transmission intensity, with over 70% of its population residing in areas of high and perennial transmission of *P. falciparum* [24]. Data on the genetic diversity of *Plasmodium* parasites in Cameroon is very limited, and none of the published work explored the host and parasite factors associated with polyclonal infection. We found a strong positive correlation between a multiplicity of infection and parasite density and that the frequency of polyclonal infection was highest in children and generally in participants with low levels of IgG antibodies against protective *P. falciparum* antigens, suggesting the dependence of both parasite density and polyclonality on the underlying host adaptive immune responses.

## 2. Materials and Methods

### 2.1. Ethical Consideration

The study was approved by the National Ethics Committee for Human Health Research of Cameroon (Ethical clearance N°: 2018/09/1104/CE/CNERSH/SP) and authorized by the Ministry of Public Health (D30-989/L/MINSANTE/SG/DROS). The field studies were also approved by local administrative authorities, including the senior divisional officer in Esse and village chiefs. Informed consent was obtained from participants over 19 years of age and from parents or legal representatives of children under the age of 19. Assent was also obtained from all children aged 12 to 19 years.

### 2.2. Study Design and Sample Collection

This study was conducted in five villages (Afanetouana (Af), Koutou (Ko), Meboe (Me), Ondoundou (On), and Ntouessong (Nt)) in the locality of Esse, Central Region of Cameroon, and belonging to the Yembouni population group [25]. The study area has been described previously [26,27], and like all communities neighboring the city of Yaounde, malaria transmission in the area is perennial and hyperendemic [24]. The blood samples were collected during a cross-sectional study conducted in November and December 2018. Participants were adults and children, excluding children under 2 years of age and pregnant women. Approximately 3 mL of venous blood was collected in EDTA-coated tubes and used for malaria diagnosis by rapid diagnostic test (RDT, Standard Q Rapid Test, SD Biosensor, Yongin-si, Republic of Korea) and for blood hemoglobin quantification using an automated hemoglobinometer (Mission Hb, San Diego, CA, USA). Axillary temperature was also recorded for each participant. All febrile cases (axillary temperature ≥ 37.5 °C) with confirmed parasitaemia by RDT were immediately treated with artemisinin-based combination therapy (ACT) according to national guidelines.

### 2.3. Detection and Quantification of Plasmodium Species

The detection and quantification of infecting *Plasmodium* species were performed by multiplex nested PCR and light microscopy, respectively [27]. Briefly, thick blood films were prepared using 5 µL of whole blood samples, followed by Giemsa staining and examination under a 100× immersion oil lens. A blood slide was declared positive when a concordant result was obtained by at least two of three independent microscopists. Slides were declared negative if no parasites were detected after a count of 500 white blood cells. Parasite density was determined based on the number of parasites per at least 200 leucocytes counted on a thick film, assuming a total white blood cell count of 8000 cells/μL of whole blood.

Genomic DNA was extracted from dried blood spots (Whatman grade 3) containing 50 µL of whole blood using the Chelex method [28] and used to screen for *Plasmodium* species infections by multiplex nested PCR targeting the 18S ssrRNA genes of *P. falciparum*, *P. ovale*, *P. malariae*, and *P. vivax*, as described by Snounou et al. [29]. For the first PCR reaction, 5 μL of genomic DNA was used in a 20 μL total volume reaction with 0.2 µM of *Plasmodium* genus-specific outer primers and 10 µL of Platinum green Hot start PCR 2X master mix (Cat: 13000014, Invitrogen, Carlsbad, CA, USA). Nested PCR was performed with 2 μL of the PCR product, 0.2 µM species-specific primers and 10 µL of Platinum green Hot start PCR 2X master mix. Both PCR steps were performed under the same cycling conditions, which included initial denaturation and enzyme activation at 95 °C for 15 min, 43 cycles of 95 °C for 45 s and 55 °C for 90 s, and a final extension at 72 °C for 5 min (Table 1). PCR assays were performed using a SimpliAmp thermal cycler (Applied Biosystems, Norwalk, Connecticut, USA), and the different *Plasmodium* species were identified on the basis of the size of the PCR product after analysis on 1.2% agarose gel under a UV transilluminator (Quantum, Vilber Lourmat, Germany). Asymptomatic infections were defined as the presence of *Plasmodium* parasites in peripheral blood, detected by multiplex nested PCR, in the absence of fever (temperature < 37.5 °C) at least 48 h before enrollment into the study. Symptomatic infections were defined as positive for *Plasmodium* infection by any one of three methods (RDT, thick smear microscopy, or multiplex nested PCR) and a history of fever (temperature ≥ 37.5 °C) within 48 h before enrollment. Participants with negative results for all three tests were considered uninfected with malaria parasites.

### 2.4. Plasmodium falciparum Genotyping Using msp2 and polyα Markers

*P. falciparum* genotyping was performed targeting two well-characterized polymorphic markers, *msp2* and *polyα,* as previously described [30,31,32,33,34,35]. The primer sequences and amplification conditions for each marker are described in Table 1.

All reactions were carried out in a final volume of 20 µL containing 200 nM of each primer, 4 µL of 5× Hot FIREpol blend master mix (Solis BioDyne, Tartu, Estonia), and nuclease-free water. In the first PCR, 5 µL of purified DNA sample was used, and in the nested reaction, 2 µL of amplicon was used. The *msp2* genotype was identified based on the size of the PCR products using a 2% agarose gel containing 0.01% of ethidium bromide and analyzed under a UV transilluminator. Identification of the *polyα* polymorphism was performed after SDS-PAGE on a 12% polyacrylamide gel. The amplification product was identified after staining the polyacrylamide gel with 0.01% ethidium bromide on a shaker at room temperature for 30 min and analyzed under a UV transilluminator. Expected heterozygosity (*He*), which is a key determinant of population structure, representing the probability of being infected by two parasite populations with different alleles at a given locus, was determined according to the formula *He* = *n*/(*n* − 1) × (1 − $\sum_{i=1}^{n}p_{i}^{2}$) with *n*: number of isolates analyzed, and *p*: allele frequency of the *i*-th allele. *He* values close to 0 indicate no genetic diversity, while an *He* value close to 1 indicates high allelic diversity [32,36]. Complexity of infection, also known as multiplicity of infection (MOI), was defined as the number of infecting genotypes per sample per genotyping method, whereas overall MOI was defined as the highest number of alleles detected by either of these genotyping methods (*msp2* or *polyα*). Samples with more than one allele (MOI > 1) were classified as polyclonal infections, whereas those with a single allele by both genotyping methods were considered monoclonal infections.

### 2.5. Anti-P. falciparum IgG Antibody ELISA

Levels of plasma IgG to four highly reactive recombinant antigens: MSP-1p19, MSP3, MSP4p20, and EBA175 (MRA-1162) [37,38], and to two soluble antigen extracts: mixed-stage antigen (anti-*Pf*) and merozoite antigen (anti-MZ) of *P. falciparum* were measured by indirect ELISA. The soluble extracts were obtained from laboratory-maintained *P. falciparum* 3D7 parasites by freeze-thaw fractionation in PBS, pH 7.4 containing protease inhibitors, whereas the *P. falciparum* Merozoite Surface Proteins were produced using a baculovirus insect cell expression system (MSP-1p19 and MSP-4p20) or in *E. coli* (MSP3) [37,39,40]. Microtiter plates (F96 CERT-Maxisorp) were coated with 100 µL of 2 mg/mL soluble antigen extract or 0.5 mg/mL recombinant antigen diluted in 0.1 M bicarbonate buffer and incubated overnight at 4 °C. After washing with PBS, pH 7.2, and blocking with 1% BSA in PBS, pH 7.2 for 1 h; the plates were further incubated for 1 h with plasma samples diluted 1/250 in PBS containing 0.05% Tween-20 and 1%BSA. Plasma from 10 unexposed European blood donors (EFS, Paris, France) was used as naive controls, while a pool of highly reactive IgG from adults living in Yaounde was used as a positive control. A goat anti-human IgG antibody conjugated to horseradish peroxidase (HRP) (1:20,000) was used as secondary antibody, and the resulting complexes were detected using TMB as a chromogenic substrate (ThermoScientific, Waltham, MA, USA). The reaction was stopped by adding 50 µL of 2N sulfuric acid solution, and optical densities (OD) were measured using a spectrophotometer at 450 nm wavelength. Data were presented as OD ratios (average OD of the sample/mean OD of unexposed samples) prior to statistical analyses.

### 2.6. Statistical Analysis

All statistical analyses were performed using Stata/MP 13.0 and GraphPad Prism 8.0.1, Harvey Motulsky, San Diego, CA, USA). Univariate and multivariate logistic regression models were used to determine the host and parasite factors associated with polyclonal infections in the study area. Only associated variables with *p* < 0.05 were included in the multivariate analysis. The Mann–Whitney test was used to compare the median between the two groups. Spearman’s test was used to determine the correlation between two quantitative parameters. Participants were classified as anemic based on WHO classification guidelines using hemoglobin levels for each age group and gender [41]. All participants <15 years of age were classified as children, whereas participants aged ≥15 years were considered adults.

## 3. Results

### 3.1. Frequency of Polyclonal P. falciparum Infection in the Study Population

Of 353 individuals tested for malaria parasites by multiplex nested PCR, 316 (89.5%) were positive for *P. falciparum* infection, of which 88.6% were *Pf* mono-infections, 8.9% were *Pf* + *Pm*, 1.9% were *Pf* + *Po*, and 0.6% were *Pf* + *Pm* + *Po*. The demographic, clinical and parasitological characteristics of the study population are described elsewhere [27]. Genotyping of the *Pf*-positive samples resulted in 250 (79.1%) positive amplifications at the *msp2* locus and 215 (68.0%) at the *polyα* locus, for a combined genotyping success rate of 88.0% (278/316). Of the 38 PCR-positive but non-genotyped samples, 23 (60.5%) were submicroscopic infections and 15 (39.5%) were low-density infections (mean parasitaemia: 273 p/µL, range: 38–1288 p/µL). The characteristics of the genotyped and non-genotyped populations are presented in Table 2.

**Figure 1:**
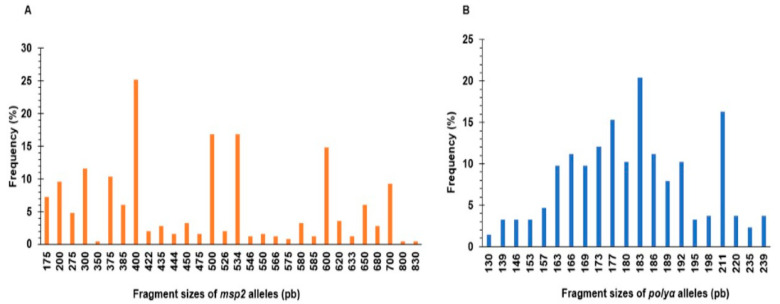
Frequency distribution of (**A**) *msp2* and (**B**) *polyα* alleles in isolates from the genotyped population. Plots show the identified allele sizes on the x-axis and their corresponding frequencies on the y-axis.

As shown in Figure 1, a total of 30 alleles appearing at frequencies between 0.4% and 25.2% were detected at the *msp2* locus, whereas 21 alleles were identified at the *polyα* locus at frequencies between 1.4% and 20.5%.

The mean number of parasite clones per infected participant (MOI) was 1.70 (range: 1–8 clones) by *msp2* genotyping and 1.67 (range: 1–6 clones) by *polyα* microsatellite analyses, for a combined MOI of 1.95 in the study population. The mean MOI was 1.58 (range: 1–5 clones) in individuals with submicroscopic infection and 2.1 (range: 1–8 clones) in the microscopy-positive subjects (*p* < 0.0001). It was 1.91 (range: 1–6 clones) in the asymptomatic individuals compared to 2.08 (range: 1–8 clones) in the symptomatic population (*p* = 0.6931), and it was 2.3 (range: 1–8 clones) in children compared to 1.60 (range: 1–5) in adults (*p* < 0.0001). The mean number of clones per infected subject varied according to locality, with Ntouessong having the highest mean MOI (MOI: 2.3, range: 1–8 clones), followed by Afanetouana (MOI: 2.1, range: 1–6 clones), Ondoundou (MOI: 1.9, range: 1–5 clones), Meboe (MOI: 1.77, range: 1–5 clones) and Koutou (MOI: 1.64, range: 1–4 clones). Expected heterozygosity (*He*) was generally high (0.54–0.91 for the *msp2* alleles and 0.58–0.87 for the *polyα* marker) in all the villages studied. The mean expected heterozygosity was 0.82 considering the *msp2* alleles and 0.85 when considering the *polyα* microsatellite marker. These results indicate a high genetic diversity of malaria parasites in the study population, with children and microscopically positive participants carrying the highest number of parasite clones.

The frequency of polyclonal infection in the study population, taking into account both allelic markers, was 60.1% (167/278), of which 80.2% were asymptomatic infections. The frequency was 37.21% in submicroscopic and 70.31% in microscopy-positive individuals (*p* < 0.0001). It was 60.36% in asymptomatic participants as against 58.93% in the febrile subjects (*p* = 0.8450). Approximately 63.47% (106/167) of individuals with polyclonal infections had parasitaemia greater than the population median, compared to only 28.82% (32/111) of high parasitaemia among individuals with monoclonal infections. The frequency of polyclonal infection varied with age and the participant’s village. It was 88.24% in children under five years old, 76.56% in children between 5–10 years old, 66.67% in 10–15 years old, and 46.85% in children and adults greater than or equal to 15 years of age. The frequency of polyclonal infection was highest in Ntouessong (74.3%), followed by Afanetouana (65.0%), Ondoundou (63.8%), Meboe (49.4%) and Koutou (47.7%). Together, these data suggest a high frequency of polyclonal *P. falciparum* infection in the study area and its probable association with age, parasite density and participant’s village.

### 3.2. Associated Factors of P. falciparum Polyclonal Infections in the Study Area

Univariate and multivariate analyses were undertaken to identify host demographic and parasite factors associated with polyclonal infection in the study area. As shown in Table 3, infection with two or more parasite clones was independently associated with age, participant’s village and parasite density.

**Figure 2:**
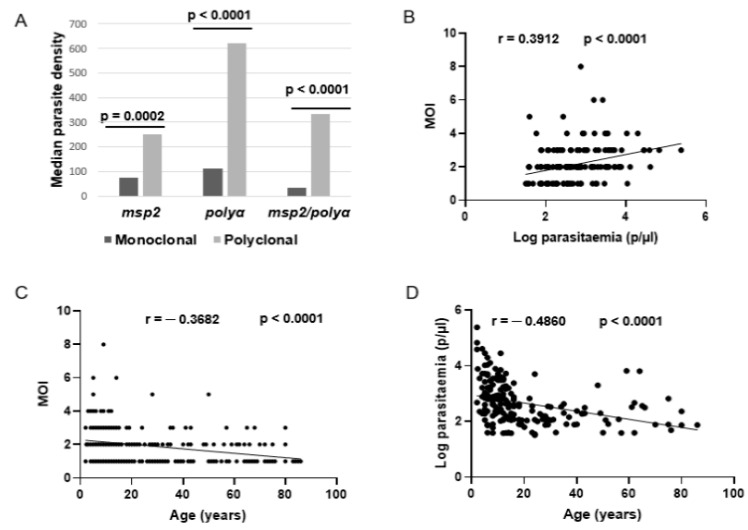
Relationship between MOI, parasitaemia and age. (**A**) Distribution of median parasite densities per infection type (monoclonal vs. polyclonal) as genotyped at each *msp2* or *polyα* locus or their combination, (**B**) Correlation between MOI and parasite density, (**C**) Correlation between MOI and age, and (**D**) correlation between parasite density and age.

Indeed, polyclonal infections were three times more likely to occur in children than adults (OR = 3.241, *p* < 0.0001) and were four times more likely to occur in participants with high parasite densities than those with low parasitaemia (OR = 4.290, *p* < 0.0001). Median parasite densities were generally higher in participants with polyclonal infections than those with monoclonal infections, and the number of parasite clones increased significantly with parasite density (r = 0.3912, *p* < 0.0001) (Figure 2). In contrast, the number of parasite clones as well as parasite densities decreased significantly with age (r = −0.3682, *p* < 0.0001 and r = −0.4860, *p* < 0.0001, respectively) (Figure 2). These findings suggest a direct link between parasitaemia and MOI and their possible influence by an age-related host factor.

Frequencies of polyclonal infection were similar between bed-net users and non-users (OR = 1.176, *p* = 0.5082), suggesting a minor role of host exposure in the acquisition of polyclonal infection. Additionally, polyclonal infections were not associated with clinical status (asymptomatic or symptomatic) or with time to previous fever episode (cf Table 3). Together, these findings suggest a lack of role of polyclonal infections in protection against clinical malaria in infected individuals.

### 3.3. Association of P. falciparum Polyclonal Infection with Underlying Acquired Immunity

**Figure 3:**
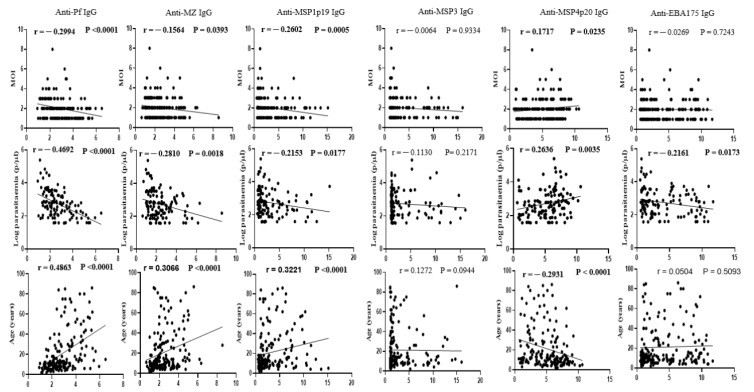
Relationship between MOI, parasitaemia, age and host adaptive immune responses. Plasma IgG levels to a *P. falciparum* mixed-stage soluble extract (anti-Pf IgG), a merozoite soluble extract (anti-MZ IgG) and to four highly reactive recombinant antigens (MSP1p19, MSP3, MSP4p20 and EBA175) were measured by indirect ELISA, and the fold changes in absorbance relative to that of non-immune plasma (*x*-axis) were correlated with obtained MOI, parasitaemia or participant age (*y*-axis).

To investigate the impact of underlying host antibody responses on the complexity of infection and frequency of polyclonal infection in the area, plasma IgG to four highly reactive *P. falciparum* recombinant antigens (MSP1p19, MSP3, MSP4p20 and EBA175) and two antigen extracts (mixed stage extract and merozoite extract) were assessed by indirect ELISA. As shown in Figure 3, the number of parasite clones per infected sample correlated negatively with IgG levels to five of these antigens (MSP1p19: r = −0.2602, *p* = 0.0005; MSP3: r = −0.0064, *p* = 0.9334; EBA175: r = −0.0269, *p* = 0.7243; anti-Pf: r = −0.2994, *p* < 0.0001; and anti-MZ: r = −0.1564, *p* = 0.0393), but correlated positively with the IgG level to the recombinant MSP4p20 protein (r = 0.1717, *p* = 0.0235).

Similarly, a negative correlation was observed between parasite density and IgG levels against the five antigens (MSP1p19: r = −0.2153, *p* = 0.0177; MSP3: r = −0.1130, *p* = 0.2171; EBA175: r = −0.2161, *p* = 0.0173; anti-Pf: r = −0.4692, *p* < 0.0001; and anti-MZ: r = −0.2810, *p* = 0.0018), whereas a positive correlation was observed for IgG levels to MSP4p20 (r = 0.2636, *p* = 0.0035). Plasma IgG levels increased with age for all five antigens (MSP1p19: r = 0.3221, *p* < 0.0001; MSP3: r = 0.1272, *p* = 0.0944; EBA175: r = 0.0504, *p* = 0.5093; anti-Pf: r = 0.4863, *p* < 0.0001; and anti-MZ: r = 0.3066, *p* < 0.0001), and decreased for the MSP4p20 antigen (r = −0.2931, *p* < 0001). Overall, these data suggest a potential role for underlying host antibody responses in limiting blood parasite densities as well as the number of infecting parasite clones per individual, with the highest parasitemia and number of clones observed generally in participants with low plasma levels of anti-*Plasmodium* IgG.

## 4. Discussion

Studies undertaken in malaria-endemic areas worldwide, particularly in areas of perennial *P. falciparum* transmission, suggest a high prevalence of polyclonal infections and their implication in protection against recurrent malaria attacks [17,19,20]. This study aimed to determine the prevalence and associated risk factors of polyclonal *P. falciparum* infections in an area of high perennial transmission and high prevalence of asymptomatic malaria parasitaemia in Cameroon. To increase the analytical sensitivity of the genotyping approach, two well-characterized allelic markers (*msp2* and *polyα*) were amplified by nested PCR, and samples were considered polyclonal if multiple alleles were detected by either one or both genotyping methods. Association of the underlying host adaptive immune responses with polyclonality (frequency and MOI) was assessed by measuring total IgG levels against several highly reactive *P. falciparum* antigens and against total soluble extracts (merozoite and mixed-stage) of a laboratory strain (*P. falciparum* 3D7 strain).

Consistent with published data on the genetic diversity of malaria parasites in hyperendemic areas [32,42,43,44,45,46], we observed a high prevalence of polyclonal infections in the study area and a positive association with parasitaemia. Approximately 29% of individuals with monoclonal infections also had parasite densities above the population median, indicating a partial or indirect relationship between parasitaemia and clone number. Indeed, both the number of clones and parasite density negatively correlated with age but were differentially associated with the sampling location, with the highest mean parasitaemia observed in Meboe while the highest mean clone number was found in Ntouessong. These findings are consistent with an independent relationship between parasitaemia and clone number and a possible role for an age-related host factor in controlling both parasitaemia and clone numbers, albeit in an independent manner.

Parasitaemia and clone number correlated negatively with IgG levels against multiple highly reactive and protective antigens of *P. falciparum*, as well as against the merozoite soluble extract. Indeed, our study is the first to report an association between the complexity of *P. falciparum* infection and anti-*Plasmodium* antibody responses. In a previous study in Nigeria [47], the authors found no correlation between IgG levels against *Pf*CSP, a circumsporozoite protein and marker of exposure to *P. falciparum*, despite a strong correlation with age. In general, with the exception of IgG levels against the MSP4p20 antigen, IgG levels increased with age, consistent with the acquisition of antimalarial immunity in long-term residents of malaria endemic zones [10,12]. The positive correlation observed between anti-MSP4p20 antibody levels and parasitaemia or MOI and the negative correlation observed with age is consistent with the recognized role of this antigen as a strong marker of *P. falciparum* infection [48,49]. Overall, our data suggest a possible implication of host antibody responses in the control of both parasitaemia and clone number in the study population. Indeed, increased levels of protective antibodies, particularly against polymorphic parasite proteins, may limit the expansion of certain clones, resulting in a lower multiplicity of infection and possibly lower parasitaemia in some individuals. In addition, anti-*Plasmodium* antibodies are generally clonotype-specific and may be short-lived [10,15,38]. Therefore, decreased levels of genotype-specific antibodies in previously exposed individuals may allow such genotypes to thrive following reinfection, resulting in polyclonal infection. 

In conclusion, the findings reported in this work are consistent with a dependence, in areas of high parasite diversity, of both parasitaemia and clonality on the degree of protective antibody immunity at the time of infection. A major drawback in this study concerns the limited sample size relative to the total population of the study area and our failure to genotype up to 12% of the PCR-confirmed infections, which may have skewed some of the factors studied. Further study of the parasite determinants of multiclonal host immunity could lead to the discovery of more effective vaccines to accelerate malaria elimination efforts globally.

## Figures and Tables

**Table 1:** Primers sequences and amplification conditions.

Locus	Primer Sequence (5′-3′)	Amplification Conditions
Species identification		
Outer	F: AGTGTGTATCAATCGAGTTTC	95 °C for 15′, 43 cycles of 95 °C for 45″ and 55 °C for 1′30″, followed by 72 °C for 5′
	R: TAACTTTCTCGCTTGCGCG	
Nested	F: AGTGTGTATCAATCGAGTTTC	95 °C for 15′, 43 cycles of 95 °C for 45″ and 55 °C for 1′30″, followed by 72 °C for 5′
	R *P.o*: TCATTCCAATTACAAAACCATG	
	R *P.m*: CCAGACTTGCCCTCCAATTGCC	
	R *P.f*: GAAAAGCTAAAATAGTTCCCC	
	R *P.v*: GTAACAAGGACTTCCAAGC	
	R *P.k*: AAGGAAGCAATCTAAGAGTTC	
*msp 2*		
Outer	F: GAAGGTAATTAAAACATTGTC	95 °C for 15′, 35 cycles of 95 °C for 45″ and 45 °C for 1′30″, followed by 72 °C for 5′
	R: GAGGGATGTTGCTGCTCCACA	
Nested	F: GAGTATAAGGAGAAGTATG	95 °C for 15′, 35 cycles of 95 °C for 45″ and 55 °C for 1′ 30″, followed by 72 °C for 5′
	R: CTAGAACCATGCATATGTCC	
*polyα*		
Outer	F: AAAATATAGACGAACAGA	95 °C for 15′, 25 cycles of 95 °C for 45″, 42 °C for 30″, 40 °C for 30″ and 72 °C for 30″, followed by 72 °C for 2′
	R: ATCAGATAATTGTTGGTA	
Nested	F: AAAATATAGACGAACAGA	95 °C for 15′, 27 cycles of 95 °C for 45″, 45 °C for 10″ and 72 °C for 30″, followed by 72 °C for 2′
	R: GAAATTATAACTCTACCA	

**Table 2:** Characteristics of the genotyped study population (N = 316).

	Genotyped (N = 278)	Non-Genotyped (N = 38)
Category	*n* (%)	*n* (%)
Age group (years)		
<5	17 (6.12)	2 (5.26)
5–10	64 (23.02)	8 (21.05)
10–15	54 (19.42)	3 (7.89)
≥15	143 (51.44)	25 (65.80)
Gender		
Male	130 (46.76)	16 (42.10)
Female	148 (53.24)	22 (57.90)
Residence		
Afanetouana	40 (14.39)	21 (55.26)
Koutou	44 (15.83)	2 (5.26)
Meboe	77 (27.70)	9 (23.69)
Ntouessong	70 (25.18)	4 (10.53)
Ondoundou	47 (16.90)	2 (5.26)
Parasitaemia		
Microscopic	192 (69.06)	15 (39.47)
Submicroscopic	86 (30.94)	23 (60.53)
Clinical status		
Asymptomatic	222 (79.86)	35 (92.11)
Symptomatic	56 (20.14)	3 (7.89)
Anemic		
Yes	65 (23.38)	14 (36.84)
No	112 (40.29)	15 (39.47)
Not Determined	101 (36.33)	9 (23.69)

**Table 3:** Factors associated with polyclonal infections in the study area.

			Univariate Analysis		Multivariate Analysis	
Parameters	Description (N)	% Polyclonal Infection (N)	OR (95% CI)	*p* Value	OR (95% CI)	*p* Value
Age group (years)	<15 (135)	74.07 (100)	3.241 (1.937–5.273)	<0.0001	1.835 (1.017–3.310)	0.044
	≥15 (143)	46.85 (67)	1			
Gender	F (148)	60.81 (90)	1.068 (0.6583–1.73)	0.7784		
	M (130)	59.23 (77)	1			
Village of residence	Afanetouana (40)	65 (26)	2.0334 (0.8445–4.691)	0.1112	1.355 (1.120–1.639)	0.002
	Koutou (44)	47.73 (21)	1			
	Meboe (77)	49.35 (38)	1.067 (0.5214–2.204)	0.8636		
	Ntouessong (70)	74.29 (52)	3.164 (1.410–6.869)	0.0040		
	Ondoundou (47)	63.84 (30)	1.933 (0.8616–4.548)	0.1220		
Length of stay in the area (years)	<15 (191)	63.35 (121)	1.686 (0.9804–2.800)	0.0504		
	≥15 (81)	50.62 (41)	1			
Bednet and/or insecticide use	No (137)	62.04 (85)	1.176 (0.7278–1.911)	0.5082		
	Yes (141)	58.16 (82)	1			
Clinical status	AS (222)	60.36 (134)	1.061 (0.5929–1.915)	0.8450		
	SY (56)	58.93 (33)	1			
Parasitaemia (p/µL) *	Low (140)	43.57 (61)	1	<0.0001	3.187 (1.762–5.766)	<0.0001
	High (138)	76.81 (106)	4.290 (2.515–7.163)			
Anemia	No (112)	58.04 (65)	1	0.1388		
	Yes (65)	69.23 (45)	1.627 (0.8673–3.067)			
Time to previous fever episode	>2 months (207)	58.45 (121)	1	0.3470		
	≤2 months (71)	64.79 (46)	1.308 (0.7532–2.295)			

* Parasite densities were considered low if less than or equal to the population median and high if greater than the median.

## Data Availability

The data presented in this study are available on request from the corresponding author.
